# Poultry: a receptacle for non-typhoidal Salmonellae and antimicrobial resistance

**Published:** 2019-02

**Authors:** Sohan Rodney Bangera, Shashikiran Umakanth, Goutam Chowdhury, Rudra Narayan Saha, Asish K. Mukhopadhyay, Mamatha Ballal

**Affiliations:** 1 Research Scholar, Enteric Diseases Division, Department of Microbiology, Kasturba Medical College, Manipal Academy of Higher Education, Manipal, India; 2 Department of General Medicine, Dr. T.M.A Pai Hospital, Melaka Manipal Medical College, Manipal Academy of Higher Education, Manipal, Karnataka, India; 3 Division of Bacteriology, National Institute of Cholera and Enteric Diseases, Kolkata, India; 4 Enteric Diseases Division, Department of Microbiology, Kasturba Medical College, Manipal Academy of Higher Education, Manipal, India

**Keywords:** Non-typhoidal salmonella, Poultry, *invA* gene, Antimicrobial resistance, Virulent genes

## Abstract

**Background and Objectives:**

Non-typhoidal Salmonellosis, a zoonotic infection associated with acute gastroenteritis is caused by non-typhoidal salmonellae (NTS). The study was carried out to determine the prevalence of NTS serovars and their antimicrobial resistance along with the presence of the virulence gene (*invA* gene) in poultry samples.

**Materials and Methods:**

This is a prospective cross-sectional study carried out at the Enteric Diseases Division, Kasturba Medical College, Manipal, South India from January 2016– December 2017. Poultry samples were collected randomly from two local poultry farms in Udupi district and processed following CDC standard protocol.

**Results:**

From the 396 poultry meat samples, intestinal contents and faecal samples collected, 58 NTS serovars were isolated showing a prevalence of 14.64%. *Salmonella* Infantis, 43.1%, 25/58 was the commonest serovar. Resistance to ciprofloxacin 72.41%, ampicillin 32.8%, gentamicin 17.24%, cotrimoxazole 29.31% and amoxicillin-clavulanic acid 6.9% was observed. The *invA* gene was detected in 43 NTS isolates (74.13%).

**Conclusion:**

Poultry sources are recognized as a significant cause for non-typhoidal salmonellosis. Therefore, hygienic measures should be initiated to reduce the contamination of meat and poultry products with virulent strains of Salmonella that are of public health significance.

## INTRODUCTION

Non-typhoidal *Salmonella* is a Gram-negative bacteria belonging to the family Enterobacteriaceae. These bacteria are said to be localized in the intestinal tract of several distinct groups of animals such as domestic fowls like chickens, ducks, geese, turkeys; farm animals like goats, cows, sheep, pigs; pets such as dogs, cats, horses, and other reptiles like turtles, lizards, snakes. They are also found in frogs, toads, rodents and other birds like parakeets, parrots and wild birds. These reservoirs of *Salmonella* can cause the disease to humans termed as non-typhoidal salmonellosis (NTS) (1, 2). Human beings obtain this infection through the ingestion of raw or undercooked contaminated food from animal origin, mainly from poultry (eggs and meat), pigs (meat) and by the consumption of unpasteurized cow milk. NTS refers to the infection produced by all serotypes of *Salmonella* except for typhoidal and paratyphoidal group. The symptoms include diarrhea, vomiting and abdominal cramps which develop 12 to 72 hours after infection. NTS have a discrete adaptation to certain animals such as *Salmonella* Choleraesuis to pigs, *Salmonella* Dublin to cattle, *Salmonella* Abortusovis to sheep and *Salmonella* Gallinarum to poultry (2, 3).

Poultry and poultry meat often get contaminated with likely pathogenic microorganisms including *Salmonella, Campylobacter, E. coli, Listeria* and *S. aureus* (3). In the poultry industry *Salmonella* and *Campylobacter* are the major foodborne pathogens. The chicken meat surface can acquire *Salmonella* from intestinal contents, fecal material or from cross-contamination during slaughtering processes (4). Chicken meat is said to be a nutritious, healthy food which is low in cholesterol and the finest source of protein in comparison with other meat. Since the chicken meat has a high moisture content, rich in nitrogenous compounds like essential amino acids, proteins, good source of minerals, vitamins and other growth factor, it serves as an ideal medium for bacterial growth as the organisms tend to remain on the surface or just under it. Both poultry muscle and skin are excellent substrates for supporting the growth of a wide variety of microorganisms. NTS being isolated from poultry sources is well documented and data are available from many parts of the world (3, 4).

NTS serovars have the ability to cause blood-stream infections when they have an assemblage of virulence genes in their salmonella pathogenicity islands (SPIs). Some of the virulence chromosomal genes of NTS are *invA, spvC, sefA, sopB* and *stn*. The invasion gene *invA,* is essential for the entry of the bacterium from the gut lumen into the epithelial cells. It is possibly responsible for the virulence of the bacteria, facilitating their entry into the blood-stream causing bacteraemia (2). The unwarranted use of antimicrobials in large-scale poultry production as veterinary medicine and also as growth promoters is an widening problem causing an increase in antimicrobial resistance in NTS and all other bacteria. The irrepressible use of antimicrobials can lead to the selection for bacterial resistance posing a threat to public health by spreading of the resistance from farm animals to the human population (5, 6). Hence, the present study was carried out to screen for the prevalence of non-typhoidal Salmonellae serovars in chickens of our district; to determine their antimicrobial susceptibility patterns and also to detect the virulence (*invA*) gene among these NTS isolates.

## MATERIALS AND METHODS

This was a prospective cross-sectional study carried out at for a period of two years (January 2016–December 2017). A total of 396 poultry meat samples, intestinal contents and faecal samples were collected randomly from two large poultry farms from Udupi district. The samples were transported and processed according to the standard protocol of WHO Global Foodborne Infections Network Laboratory Protocol (7).

### Isolation and identification of NTS from fecal samples.

About 20 gms of the freshly passed fecal material or intestinal contents were collected in a clean container. These were then transferred into 200 ml buffered peptone water and incubated at 37°C for 18 hours. 1 ml of the broth was transferred to 10 ml tetrathionate broth and further incubated at 37°C for 24 hours. A loopful of the broth was plated onto *Salmonella* differential agar (Hi ChromeTwin Pack, RajHans Medium M1078) and incubated at 37°C for 18–24 hours. Colonies which appeared pink-red in color were subjected to further analysis of biochemical tests for the identification of NTS.

### Antigenic profiling.

*Salmonella* isolated were serotyped with specific polyvalent O and H antisera (Difco™ Antiserum Solutions - Becton Dickinson). Non-typhoidal *Salmonella* strains confirmed were further subjected for serovar identification and its antigenic profiling was done at the national reference center - National Institute of Cholera and Enteric Diseases (NICED), Kolkata, India.

### Antimicrobial susceptibility test.

Antimicrobial susceptibility testing was done for various antimicrobials including amikacin (30 μg), ampicillin (10 μg), amoxiclav (30 μg), azithromycin (15 μg), ceftazidime (30 μg), ceftriaxone (30 μg), cefuroxime (30 μg), chloramphenicol (30 μg), ciprofloxacin (5 μg), co-trimoxazole (25 μg) and gentamicin (10 μg). *Escherichia coli* 25922 was used as the standard control strain. The antimicrobial susceptibility testing was performed by modified Kirby-Bauer's disk diffusion method according to Clinical Laboratory Standards Institute guidelines (8).

### Detection of virulence-specific gene – *invA* gene of NTS.

*Salmonella entericasero* type (4, 5, 12: i:-) was used as the positive control and NTS (n=58) genomic DNA were extracted using the boiling method (9). The extracted DNA was quantified using Nanodrop (Eppendorf BioPhotometer D30) with absorbance values at 320 nm. The primer for the *invA* gene was designed using the primer 3 output sequence (available at http://bioinfo.ut.ee/primer3-0.4.0/). The forward primer of the *invA* gene GTTTACGACCT-GAATTACTG and reverse primer GATAAGAC-GACTGGTACTGA with a base pair of 239 was used in this study. The PCR reaction was carried out as per Mir et al. (10) in a total volume of 12.75 μl, consisting of primers (0.5 μl each), 5 ng of DNA (1 μl), Taq polymerase master mix (6.25 μl) (Go Green, Promega Corporation, USA) and sterile Milli-Q water was added to make the final volume. The PCR reaction consisted of 1 cycle of an initial denaturation of 94°C for 2 min, followed by 35 cycles of 95°C for 1 min, 57°C for 1 min, and 72°C for 2 min and a final extension cycle was performed at 72°C for 10 min. The amplified products were analyzed in a 2.5% (w/v) agarose gel in 1× Tris base, acetic acid and EDTA (TAE) buffer. Ethidium bromide (Sigma-Aldrich, USA) (0.5 μg/mL TAE) stained DNA amplicons were seen using a gel imaging system (Biotron Healthcare).

## RESULTS

**Fig. 1.**
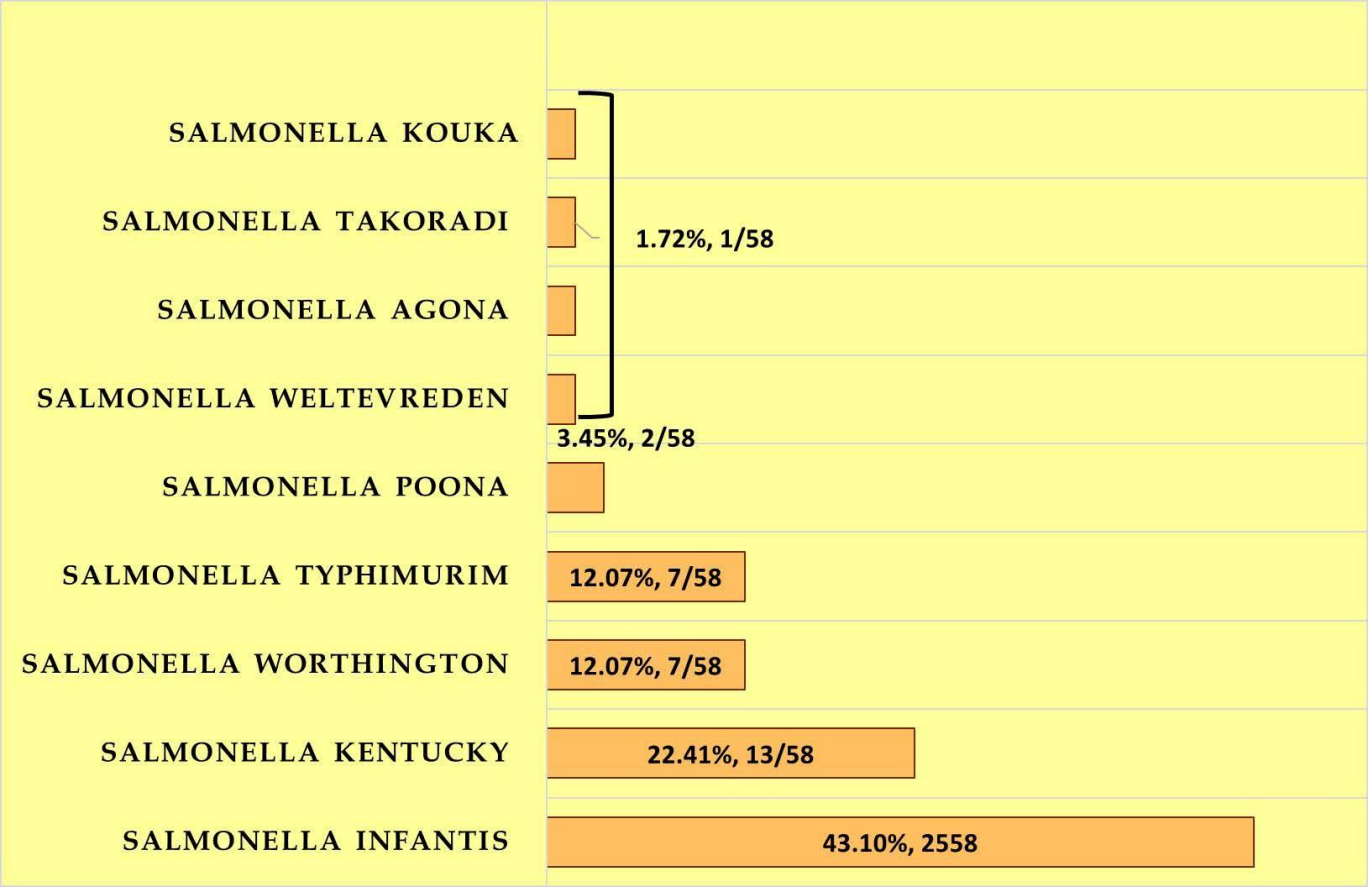
Non-typhoidal *Salmonella* serovars isolated from poultry

Of the 396 poultry meat samples, intestinal contents and faecal samples collected, 58 NTS serovars were isolated showing a prevalence of 14.64%. *Salmonella* Infantis, 43.10% (25/58) was the predominant serovar isolated followed by *Salmonella* Kentucky, 22.41% (13/58). *Salmonella* Poona (2/58, 3.45%) and *Salmonella* Kouka (1/58, 1.72%) are the NTS serovars first time isolated in our country either from human or food animal reservoirs according to PubMed search. NTS serovars isolated from poultry are depicted in (Fig. 1.)

**Fig. 2.**
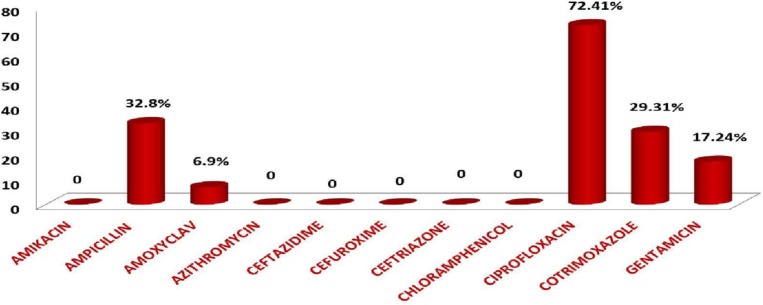
Antimicrobial resistance pattern of non-typhoidal *Salmonella* isolates

The antimicrobial resistance pattern showed resistance to ciprofloxacin in 72.41%, ampicillin in 32.8%, gentamicin in 17.24%, cotrimoxazole in 29.31% and amoxicillin-clavulanic acid in 6.9%. Fig. 2. depicts the antimicrobial resistance pattern.

**Fig. 3.**
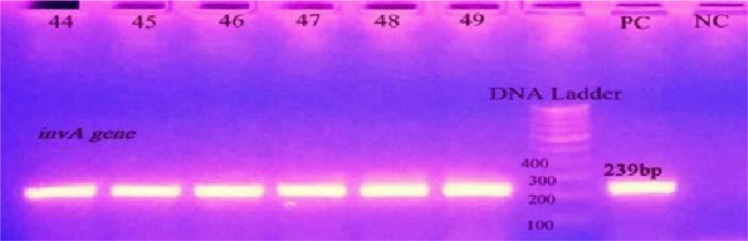
Gel image showing amplification of the *invA* gene. bp: base pair, PC: Positive control, NC: Negative control

Of the 58 NTS serovars, the *invA* gene detected in 43 NTS isolates showing a proportion of 74.13%. Fig. 3. shows the bands which are positive for the *invA* gene. Table 1 shows the NTS serovars with the proportion of the *invA* gene present in them.

**Table 1.** NTS serovars with *invA* gene

**NTS Serovars**	***invA* gene**
*Salmonella* Infantis	16/25, 64%
*Salmonella* Kentucky	10/13, 76.92%
*Salmonella* Worthington	7/7, 100%
*Salmonella* Typhimurium	7/7, 100%
*Salmonella* Poona	2/2, 100%
*Salmonella* Agona	0
*Salmonella* Weltevreden	1/1, 100%
*Salmonella* Takoradi	1/1, 100%
*Salmonella* Kouka	0

## DISCUSSION

One of the typical poultry found all over the world is chicken. The term broiler is applied for chickens since they are bred for meat and also because of their speedy growth. ICRA (a renowned Indian independent and professional investment information and credit rating agency) report of 2014 states that the growth rate of the Indian poultry sector is 8–10 percent annually over the last decade (5). Some by-products of antibiotic production by the pharmaceutical industry comprise of a high level of vitamin B12 which when fed to poultry animals results in higher growth rate, an example of such by-product of antibiotic production is dried *Streptomyces aureofaciens* broth. Thus, the antibiotics are incorporated into the feed given to the poultry for enhanced growth production (11). When an animal is treated with an antimicrobial drug, bacteria which are sensitive to the antimicrobials are eliminated while bacteria resistant to these antimicrobial drugs survive and replicate in the host increasing their population. This poses a major risk for humans due to the acquisition of antibiotic resistance in the bacteria harboring in the poultry. This can give rise to the emergence of resistant bacteria in the animal which can be transmitted to the humans through food, environment and also via direct contact with farm animals (6). This causes an obstruction in the treatment of human diseases causing difficulty in treatment. In addition, these bacteria can transfer their resistance genes to a sensitive strain through gene transfer. Thus, antimicrobials can play a vital role in increasing the prevalence of resistant bacteria among human pathogens and animal pathogens and also with their respective normal bacterial flora (12).

Antibiotic growth promoters (AGP) are defined as any medicine when administered at a low, sub-therapeutic dose destroys or inhibits bacteria. These AGPs are given along with the feed at a minimal dose on an even basis. AGPs can be differentiated from therapeutic and prophylactic antibiotic use as the latter being delivered at higher doses and usually introduced through the water. Antibiotic growth promoters are said to suppress the lining of the normal bacterial flora of the gut causing accumulation of nutrients for the chicken to absorb resulting in greater weight gain. Research reveals the benefits of AGPs are significant and have been helpful in the case of sick birds and birds which are housed in cramped and unhygienic condition (5, 6, 12). In India, the commonly used AGPs in the poultry industry include oxytetracycline, chlortetracycline, bacitracin, furazolidone, enrofloxacin, cephalosporins, ciprofloxacin and tylosin. The other AGPs premix used are tiamulin hydrogen fumarate, tylosin phosphate, oxytetracycline, tylosin tartrate, enramycin and virginiamycin. The reason for the higher mortality rate in chickens is due to the occurrence of bacterial diseases like acute gastroenteritis, septicemia caused by *Salmonella* and *E. coli*, a chronic respiratory disease caused by *Mycoplasma* and necrotic enteritis caused by *Clostridium*. In order to prevent these diseases and infections, antibiotics are being administered as a necessary measure for treatment and prevention. The choice, period and frequency of antibiotic depend on the farm hygiene, sanitation, infrastructure, veterinary guidance, the experience of the poultry owner and the nature of diseases prevalent in the area. Veterinarians have practically no role in the introduction of antibiotics or AGPs as the nontherapeutic dose for poultry (6).

In the present study, NTS isolates have shown resistance to ampicillin in 32.8%, cotrimoxazole in 29.31%, gentamicin in 17.24% & amoxicillin-clavulanic acid in 15.38%. According to our study a greater level of resistance to ciprofloxacin (72.41%) was observed. Similar studies were done in Kashmir by Mir et al. has shown ciprofloxacin resistance about 71.42%, this shows that level of resistance in the country tends to be more or less similar (10). In a study carried out in Japan, resistance to ciprofloxacin was only 13.3% (13) and in Palestine, ciprofloxacin resistance was 45% (14). In Nigeria, high frequency of resistance (81%) was observed for ciprofloxacin (15). Enrofloxacin is a synthetic fluoroquinolone antimicrobial agent administered orally to chickens, for the treatment of respiratory and alimentary tract infections. However, as ciprofloxacin is available at a low cost, most of the poultryfarm owners tend to use it, thus an antibiotic or meant not developed for animal use is being utilized. The unlicensed version without the seal is available at multiple veterinary centers in India. These imported versions are materialized from China (6). In the present study, multidrug resistance was seen in 13.8% of the NTS isolates showing resistance to ampicillin, amoxiclav, ciprofloxacin, norfloxacin, gentamicin and trimethoprim-sulfamethoxazole. A study carried out in the Philippines revealed that all of the isolates were sensitive to norfloxacin, gentamicin and cephalothin, however, all of the isolates remained resistant to nitrofurantoin. A study by Balala et al. reported Multidrug resistance in 9.7% of the NTS isolates showing resistance to nitrofurantoin, tetracycline and trimethoprim-sulfamethoxazole (16). A study in south India shows all the isolates resistant to oxytetracycline since the same antibiotic is used as an AGP for poultry in the same state. The other inherited resistance observed in the study were tetracycline (63.4%), nalidixic acid (63.4%) and streptomycin (61%) (17). Data from our study reveals that there is an extremely high rate of quinolone-resistant NTS (72.41%). This occurrence of resistance may be due to the consequences owing to the administration of fluoroquinolones to poultry. This is implemented along with the feed in various farms of our district. Ciprofloxacin is the first-line drug generally prescribed for non-typhoidal salmonellosis while cotrimoxazole, moxifloxacin, levofloxacin andcephalosporins like ceftriaxone and cefotaxime are alternatives. Resistance to ceftriaxone was not detected in any of these NTS isolates. Thus, presently ceftriaxone is the alternative therapy for patients being infected with multidrug-resistant NTS through food animals (14).

The overall prevalence of NTS isolated from our district is 14.64% which is concordant with the study done in north-east India which showed the prevalence of 14.7% (18). Although the prevalence rate observed in our study was higher when compared with the studies done in the government poultry farms of Kashmir valley of northern India where it reports the prevalence as 6.88% (10). The prevalence rate is also ten times more than the study done by a neighboring state in southern India which reported 1.73% (17). Prevalence of the NTS from poultry varies from country to country and continent to continent. Studies done in south-east Asian countries, such as Philippines showed a prevalence rate of 4.9%NTS from poultry (16), and Vietnam showed 45.6% (19). Jordan showed the highest prevalence of 66% (20), farm prevalence of Nigeria was 43.6% (21), Japan was 54% (13), Egypt was 14.3% (22), Iraq was 10.39% (23) and the United States of America showed 12.4% (24). The occurrence of *Salmonella* serovars differed among different states of our country where the investigation was done. The predominant serovar isolated in our study was *Salmonella* Infantis (25/58, 43.1%) followed by *Salmonella* Kentucky (11/58, 18.97%) which is in contrary to the studies done in north India which reports *Salmonella* Gallinarum *as* the predominant serovar whereas in north-east India, *Salmonella* Gallinarum and *Salmonella* Typhimurium are the commonest serovar from poultry (10, 18). The isolation rate of *Salmonella* Typhimurium from poultry sourceswas 12.07% (7/58) in our study which is one of the significant NTS serovar causing human non-typhoidal salmonellosis in this region. Report suggests *Salmonella* Gallinarum was the aetiology for the three different outbreaks of salmonellosis in different farms in Kerala, south India in the year 2005 (25). Nigeria reports *Salmonella* Virchow as the commonest serovar isolated from poultry (15). *Salmonella* Weltevreden was the predominant serovar isolated in a study done in Philippines (16). Only one *Salmonella* Weltevreden (1.72%) was isolated in our study. Japanese study states that *Salmonella* Infantis (33%) was the most common serotype isolated from the samples of chickens which are in concordant with the findings of our study (13). *Salmonella* Kentuckyis the commonest serovarat the United States of America, in our study it is the second commonest serovar after *Salmonella* Infantis (24). The variation in the serovars isolated differs with regard to the continents, countries and geographical regions. This variability is based on the grounds like environmental contamination, control system, contamination and methods of sampling (21).

NTS stands as a facultative intracellular pathogen, this ability is due to the presence of a cluster of genes in the *Salmonella* pathogenicity islands (SPIs) positioned in the bacterial chromosome and plasmids. There are approximately 60 virulence genes in SPIs. *invA, spvR, spvC, fimA* and stnare some of the virulence genes of NTS. *invA* gene is a unit of the SPI 1 which encodes a protein in the inner membrane of bacteria which is responsible invasion to the epithelial cells of the host (2). The PCR assay for the detection of *invA* gene showed a proportion of 74.13% (43/58) which is lesser in comparison with other studies carried out where *invA* gene was present in all the NTS isolates (10, 26). A study done by Smith et al. in Nigeria showed the PCR analysis of the NTS isolates for the *invA* gene was 96.1% (27). A proportion of *invA* gene of 55% among NTS poultry isolates was also observed in a study done by Sharma et al., in northeast India (28).

In our study, seventy-four percent of the NTS serovars were positive for the presence of *invA* gene which codes for invasion and survival in macrophages and in the internalization needed for the invasion of deeper tissue there by causing invasive infections. Research carried out also proposes that the primary food consumed by the patients suffering from non-typhoidal salmonellosis was found to be poultry products such as chicken meat and eggs (72.7%) (2). Centers for Disease Control and Prevention (CDC) as of July 13, 2018, reported multistate outbreaks of NTS linked to contact with live poultry. This outbreak resulted in 212 affected with non-typhoidal salmonellosis in 44 states and 34 hospitalizations (29). Centers for Disease Control and Prevention (CDC) has reported a total of 53 live poultry–associated salmonellosis (LPAS) outbreaks during the year 1990–2014, involving 2,630 illnesses, 387 hospitalizations, and 5 deaths in the Unites states (29).

Poultry is said to be an important source of NTS. The poultry samples in our study were contaminated with NTS isolates. These were positive for the presence of *invA* virulence gene (74.13%). NTS isolates have shown an increased resistance to quinolones, penicillins, cotrimoxazole and beta-lactamase inhibitors due to the inappropriate use of antibiotics in food animals contributing to the increase in antimicrobial resistance. These resistant and virulent strains can directly infect humans through food causing invasive infections. Managing the public health threat posed by antimicrobial resistance requires effective antimicrobial surveillance programmes, proper food handling practices and prudent use of antibiotics in the poultry. We propose that non-antibiotic growth promoters such as herbal supplements that are efficient and cost effective be administered to the poultry substituting antibiotics.
